# System-Level, Molecular and Cellular Mechanisms of Selected Plant Adaptogens—A Review

**DOI:** 10.3390/nu18060931

**Published:** 2026-03-16

**Authors:** Sebastian Such, Czesław Puchalski, Łukasz Kogut, Grzegorz Zaguła

**Affiliations:** Department of Bioenergetics, Food Analysis and Microbiology, Institute of Food Technology and Nutrition, Faculty of Technology and Life Science, University of Rzeszów, Aleja Rejtana 16C, 35-959 Rzeszow, Poland; fenikstvit@gmail.com (S.S.); cpuchal@ur.edu.pl (C.P.); lukasz.kogut2@wp.pl (Ł.K.)

**Keywords:** adaptogens, molecular mechanisms, HPA axis, Hsp70, neuroprotection, chronic stress, ginsenosides, withanolides, salidroside, bacosides

## Abstract

**Background/Objectives**: Adaptogens are plant-derived substances that enhance the body’s nonspecific resistance to physical, chemical, biological, and psychological stressors by normalizing physiological functions. This article discusses the molecular mechanisms of action of seven key plant adaptogens—*Rhodiola rosea*, *Schisandra chinensis*, *Withania somnifera*, *Eleutherococcus senticosus*, *Panax ginseng*, *Ocimum tenuiflorum*, and *Bacopa monnieri*—in the context of chronic stress and lifestyle-related diseases. **Methods**: A review of the scientific literature is performed, including preclinical *in vitro* and *in vivo* studies, randomized placebo-controlled clinical trials, and studies employing network pharmacology analyses, molecular docking, and genomic techniques such as gene expression profiling. The interactions of active constituents with signaling pathways, molecular targets, and synergistic mechanisms were analyzed based on publications from the years 2010–2025. **Results**: Adaptogens exhibit pleiotropic activity: they regulate the HPA axis (Hypothalamic–Pituitary–Adrenal axis); induce Hsp70/Hsp16 expression; modulate SAPK/JNK, FOXO, and NF-κB pathways; and demonstrate antioxidant and mitoprotective effects. Specific mechanisms include: salidroside from *R. rosea* activating PI3K/Akt; schizandrin B from *S. chinensis* stimulating Hsp70; withanolides from *W. somnifera* inhibiting PDE4D; ginsenosides from *P. ginseng* suppressing FKBP51; and bacosides from *B. monnieri* enhancing acetylcholine synthesis. Clinical studies confirm reductions in cortisol levels (14–30%), decreased fatigue, and improved cognitive function without adverse effects. **Conclusions**: Understanding the molecular mechanisms of adaptogens supports their application in integrative medicine for the treatment of stress-related disorders, depression, anxiety, and neurodegenerative diseases. Further clinical studies are needed to optimize dosages and standardize extracts.

## 1. Introduction

Adaptogens constitute a group of plant-derived substances that have been used for thousands of years in traditional medical systems, such as Ayurveda and Traditional Chinese Medicine, to enhance the body’s resistance to various stressors [1]. The term “adaptogen” was first defined by the Russian toxicologist Nikolai Lazarev in the 1950s–1960s, who described these substances as compounds capable of increasing the organism’s “state of nonspecific resistance” [2]. This definition was later refined by Brekhman and Dardymov, who in 1969 proposed that adaptogens are non-toxic substances exerting a nonspecific stimulatory effect that enhances the body’s resistance to physical, chemical, biological, and psychological stressors while producing a normalizing effect regardless of the direction of prior pathological changes [1,2].

The adaptogenic plants selected for this article were not chosen at random. They represent the most extensively studied species with a well-established position in both traditional medical systems and contemporary phytotherapy while simultaneously differing in their chemical profiles and spectrum of biological activity [1,2].

From a botanical and agronomic perspective, the discussed species represent diverse climatic zones and distinct cultivation requirements. *Rhodiola rosea* occurs primarily in mountainous and Arctic regions, preferring cold climates and well-drained soils. In contrast, *Withania somnifera* and *Ocimum sanctum* are native to subtropical and tropical climates, requiring higher temperatures and longer growing seasons. *Panax ginseng* and *Eleutherococcus senticosus* are cultivated mainly in East Asia, where soil conditions, humidity, and a multi-year growth cycle play a crucial role in the accumulation of bioactive compounds [1,2].

The chemical composition of these adaptogens is equally diverse. The most important groups of bioactive constituents include triterpenoid saponins (ginsenosides in ginseng, eleutherosides in eleuthero), lignans (schisandrins in *Schisandra chinensis*), phenylpropanoids (salidroside and rosavins in *Rhodiola rosea*), withanolides in ashwagandha, eugenol and phenolic acids in holy basil, and bacosides in *Bacopa monnieri*. This phytochemical diversity translates into distinct—although partially overlapping—molecular mechanisms of action, which justifies their joint analysis in the context of the organism’s adaptive response. The selection of these species enables a comparative evaluation of adaptogens representing different phytochemical classes, diverse medical traditions, and distinct biological models of action [1,2].

In recent decades, adaptogens have attracted significant scientific interest due to their potential to mitigate the effects of chronic stress, which is considered one of the major risk factors for many civilization-related diseases, including metabolic disorders, cardiovascular diseases, depression, and cognitive impairment [3]. Unlike conventional stimulants, such as sympathomimetics, adaptogens do not cause dependence, tolerance, or energy depletion, and their effects are particularly pronounced under conditions of fatigue and stress [1]. The mechanism of action of adaptogens fundamentally differs from that of traditional drugs, which typically act on single molecular targets. Adaptogens exhibit pleiotropic therapeutic activity, simultaneously modulating multiple signaling pathways and molecular targets [3].

Key molecular mechanisms underlying the action of adaptogens include regulation of the hypothalamic–pituitary–adrenal (HPA) axis, stimulation of heat shock protein expression (Hsp70, Hsp16), modulation of stress-activated protein kinases (SAPK/JNK), effects on transcription factors (FOXO, NF-κB), as well as antioxidant and mitoprotective activities [1,2]. Studies employing network pharmacology, molecular docking, and genomic techniques such as gene expression profiling have revealed complex interaction networks between the active constituents of adaptogens and multiple molecular targets involved in adaptive stress responses [3,4].

The aim of this article is not only to provide a concise synthesis of the current state of knowledge regarding the mechanisms of action of selected adaptogenic plants, but above all to offer a critical analysis of their molecular targets and to identify both shared and distinct signaling pathways responsible for the adaptive effect.

In contrast to previous studies, which have primarily focused on clinical outcomes or individual biological pathways, the present work places particular emphasis on an integrative perspective of adaptogen activity within the neuroendocrine–immune axis, taking into account potential mechanisms of synergy and antagonism among their active constituents [5].

Such an approach enables a more precise understanding of their role in modulating the stress response and highlights future research directions in the fields of integrative medicine and systems pharmacology [3].

## 2. Materials and Methods

This review was prepared on the basis of a systematic search and analysis of scientific literature available in the PubMed, Scopus, Web of Science, and Google Scholar databases, covering publications from 2010 to 2025. The search strategy included keywords such as “adaptogens”, “molecular mechanisms”, “HPA axis”, “Hsp70”, “neuroprotection”, “ginsenosides”, “withanolides”, “salidroside”, and “bacosides”, used both individually and in combination with the names of the following plants: *Rhodiola rosea*, *Schisandra chinensis*, *Withania somnifera*, *Eleutherococcus senticosus*, *Panax ginseng*, *Ocimum tenuiflorum*, and *Bacopa monnieri*.

The analysis included preclinical studies (*in vitro* and *in vivo*), randomized placebo-controlled clinical trials (RCTs), meta-analyses, studies in the field of network pharmacology, molecular docking studies, and gene expression profiling analyses, with particular emphasis on the pleiotropic mechanisms of action of adaptogens at the molecular and cellular levels.

Interactions between bioactive compounds and key signaling pathways (including PI3K/Akt and Nrf2/NF-κB), target proteins (e.g., FKBP51, CRF1), and gene regulatory networks were analyzed using bioinformatic tools commonly applied in network pharmacology research. These approaches included protein–protein interaction (PPI) analysis, molecular docking, and the evaluation of transcriptomic datasets available in the literature.

The analysis was integrative in nature and involved the synthetic interpretation of published findings in order to model potential synergistic and multi-target effects of adaptogens. No experimental studies were conducted within the framework of the present work; all data were derived from the reviewed scientific literature.

## 3. Mechanisms of Action of Selected Adaptogens

The adaptogens discussed in this section were selected to represent distinct dominant molecular mechanisms involved in stress adaptation. Although all of them share pleiotropic activity and converge on common signaling pathways, each plant demonstrates a characteristic primary molecular target (e.g., mitochondrial protection, glucocorticoid receptor modulation, cholinergic regulation). Therefore, the following subsections analyze each species in a unified framework to highlight both shared and specific mechanisms.

### 3.1. Rhodiola rosea L.

**Figure 1:**
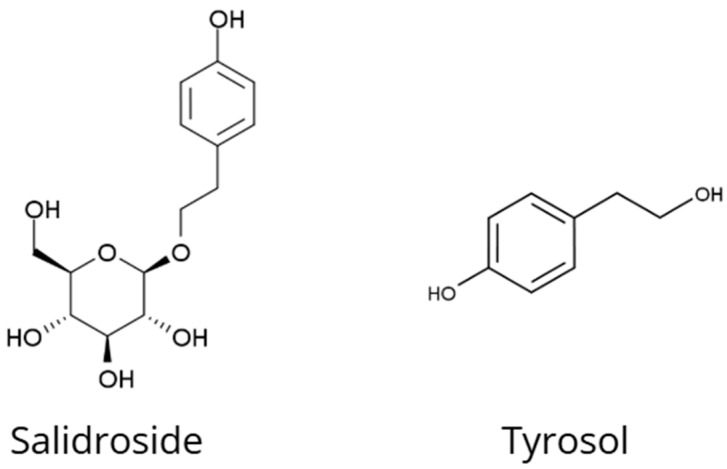
Chemical structures of salidroside and tyrosol, two characteristic phenolic compounds occurring in *Rhodiola rosea*.

*Rhodiola rosea* L. is an adaptogen traditionally used in Russian and Scandinavian medicine to enhance resistance to stress, improve physical and mental endurance, and alleviate fatigue [1,6]. Salidroside, a phenylethanoid glycoside that constitutes the principal active compound of *R. rosea* extracts, exhibits a broad spectrum of neuroprotective activity (Figure 1) [7,8]. Salidroside stimulates the central nervous system through modulation of the PI3K/Akt/GSK-3β signaling pathway, leading to increased survival of hippocampal neurons in models of diabetes and cerebral ischemia [7,9].

Salidroside protects neurons against toxic damage induced by sodium azide (NaN_3_), glutamate, and H_2_O_2_ through activation of the antioxidant Nrf2 pathway and preservation of mitochondrial respiratory chain complex I activity [8]. This mechanism involves nuclear translocation of Nrf2 and upregulation of antioxidant enzymes such as superoxide dismutase (SOD), catalase, and glutathione peroxidase (GSH-Px), thereby preventing ROS accumulation and apoptosis [8]. In addition, salidroside promotes peripheral nerve regeneration by stimulating the proliferation and functional activity of Schwann cells and by increasing the expression of neurotrophins, including BDNF, GDNF, and CNTF [10,11].

Tyrosol, another phenylethanoid metabolite of *R. rosea*, enhances the phosphorylation of endothelial nitric oxide synthase (eNOS) and the transcription factor FOXO3a, which represent key molecular targets in adaptogenic mechanisms [1]. Tyrosol induces the expression of sirtuin 1 (SIRT1), a protein associated with longevity, thereby strengthening antioxidant responses and mitochondrial protection [1]. Activation of SIRT1 by tyrosol modulates pathways related to autophagy and mitochondrial biogenesis, preventing cellular aging and stress-induced damage [1].

Rosavins (rosavin, rosin, and rosarin), triterpene glycosides specific to *R. rosea*, exhibit synergistic effects with salidroside in modulating the hypothalamic–pituitary–adrenal (HPA) axis through interactions with glucocorticoid receptors and the JNK signaling pathway [1,6]. This combination suppresses excessive cortisol and nitric oxide production under stress conditions, thereby restoring neuroendocrine homeostasis [1]. Rosavins also increase ATP levels and modulate proteasome activity, promoting the clearance of pathological proteins such as α-synuclein [12].

Randomized clinical trials have demonstrated that *R. rosea* (200–680 mg/day of extract) significantly reduces fatigue symptoms in patients with occupational burnout and improves cognitive function in individuals exposed to psychological stress [1]. In double-blind, placebo-controlled studies, *R. rosea* reduced salivary cortisol levels by 14–30% after four weeks of supplementation while simultaneously improving attention, working memory, and reaction speed [1]. Meta-analyses confirm a moderate adaptogenic effect of *Rhodiola rosea* in reducing fatigue (SMD = −0.69) and improving stress-related parameters without significant adverse effects [1].

Additionally, *R. rosea* modulates immune function by increasing the activity of natural killer (NK) cells and T lymphocytes, supporting its adaptogenic effects under conditions of stress-induced immunosuppression [6]. Animal studies have demonstrated protection against cerebral ischemia via regulation of the PI3K/PKB/Nrf2/NF-κB pathway, independent of complement component C3 activity [13]. These multitarget mechanisms provide a mechanistic basis for the clinical efficacy of *R. rosea* in the treatment of anxiety disorders, depression, and post-traumatic stress disorder (PTSD) [1].

### 3.2. Schisandra chinensis (Turcz.) Baill.

**Figure 2:**
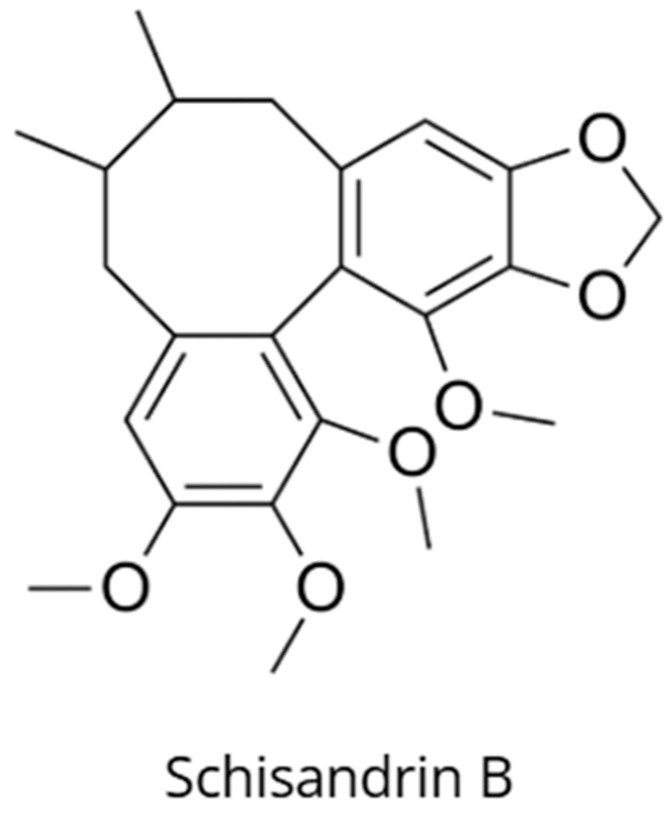
Chemical structure of schisandrin B, a representative lignan and one of the principal bioactive constituents of *Schisandra chinensis*.

*Schisandra chinensis* (Turcz.) Baill., known as *wu wei zi* in Traditional Chinese Medicine, is an adaptogen containing more than 50 dibenzocyclooctadiene lignans, among which schisandrin B (Sch B) is the principal active constituent (Figure 2) [14,15]. Schisandrin B exhibits potent neuroprotective effects associated with the stimulation of heat shock protein 70 (Hsp70) expression in healthy neuronal cells [1,14]. This hormetic effect of schisandrin B is based on an adaptive enhancement of cellular resistance to oxidative stress via Hsp70 induction, thereby preventing apoptosis and neuronal degeneration in models of aging and neurodegenerative diseases [1,14].

The stimulation of Hsp70 by schisandrin B is linked to improvement of mitochondrial glutathione status (mtGSH), increased activity of antioxidant enzymes, and enhanced ATP production [1,14]. Schisandrin B alleviates age-related impairment of mitochondrial oxidative capacity by protecting complexes I and II of the electron transport chain from oxidative damage [1]. In Alzheimer’s disease models, schisandrin B inhibits β-amyloid accumulation through suppression of the RAGE/NF-κB/MAPK signaling pathway and by increasing the expression of Hsp proteins and beclin-1, key markers of autophagy [16].

*S. chinensis*, including schisandrin B, influences the PI3K/AKT/mTOR signaling pathway, modulating autophagy and conferring protection against atherosclerosis through the reduction of oxidative stress and inflammation [17,18]. Schisandrin C from *S. chinensis* attenuates indomethacin-induced intestinal injury via this pathway, decreasing apoptosis and improving intestinal barrier integrity [19]. In Huntington’s disease models, *S. chinensis* seed extracts reduce disease symptoms by activating Nrf2 and inhibiting NF-κB/MAPK signaling, thereby limiting microglial migration [20].

Network pharmacology studies have demonstrated that *S. chinensis* exerts its effects through the AGEs/RAGE pathway, leading to attenuation of inflammatory responses in diabetic kidney disease (DKD) [21]. A mixture of *S. chinensis* fruits modulates the expression of RAGE, NF-κB, and pro-inflammatory cytokine genes, as confirmed by molecular docking and experimental validation [21]. This mixture improves renal function by reducing fibrosis and oxidative stress in DKD models [21].

Schisandrin B protects dopaminergic neurons against 6-hydroxydopamine (6-OHDA)-induced toxicity by limiting endoplasmic reticulum stress related to protein misfolding and by activating sirtuin 1 (SIRT1) [22]. In oxidative stress models, schisandrin B increases the survival of PC12 cells through inhibition of NF-κB and activation of Nrf2 signaling [16]. Clinically, *S. chinensis* extracts have been shown to improve cognitive performance and reduce fatigue in randomized clinical trials [14].

*S. chinensis* also exhibits cardioprotective effects mediated by its lignans, including schisandrin B, which reduce oxidative stress and inflammation in myocardial tissue [23]. In obesity models, schisandrin B prevents skeletal muscle atrophy through regulation of the AMPK/mTOR pathway [24]. Collectively, these findings underscore the pleiotropic adaptogenic properties of *Schisandra chinensis* [3].

### 3.3. Withania somnifera (L.) Dunal

**Figure 3:**
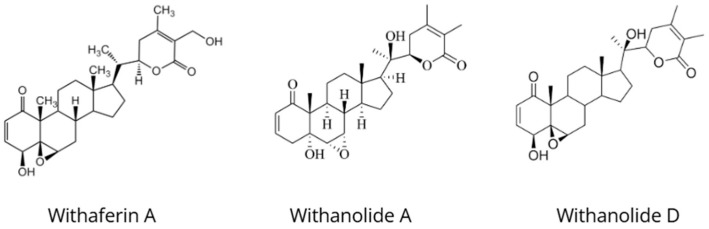
Chemical structures of withaferin A, withanolide A, and withanolide D, representative withanolide compounds occurring in *Withania somnifera*.

*Withania somnifera* (L.) Dunal, commonly known as ashwagandha, Indian ginseng, or winter cherry, is one of the most important medicinal plants in Ayurvedic medicine. It has traditionally been used as a *rasayana* (rejuvenating tonic) to promote vitality, stress resilience, and longevity (Figure 3) [25,26]. Ashwagandha contains withanolides as its principal bioactive constituents responsible for adaptogenic activity, including withaferin A, withanolide A, withanolide D, and sitoindosides, which exhibit broad and multitarget pharmacological effects [27,28,29]. Withanolides are steroidal lactones with an ergostane skeleton composed of 28 carbon atoms, derived from the mevalonate pathway and steroid biosynthesis [30].

Withaferin A, the most extensively studied withanolide, displays potent neuroprotective, anticancer, anti-inflammatory, and stress-modulating properties through its effects on multiple signaling pathways [27,31,32]. It acts in part via regulation of the PI3K/Akt pathway, leading to increased expression of the anti-apoptotic protein Bcl-2 and inhibition of apoptotic processes in neuronal cells and cardiomyocytes [32,33]. In models of diet-induced hypercholesterolemia and atherosclerosis, withaferin A reduces oxidative stress and inflammatory markers (TNF-α, IL-6, NF-κB) through modulation of PI3K/Akt signaling, thereby protecting vascular endothelial function [33].

In addition, withaferin A targets the protein mortalin, resulting in activation of the tumor suppressor protein p53 and mitochondrial dysfunction in cancer cells [31,32]. Mortalin (mitochondrial HSP70) binds and sequesters p53 in the cytoplasm, thereby preventing its tumor-suppressive activity. Withaferin A disrupts the mortalin–p53 interaction, restoring p53 function and inducing apoptosis in cancer cells [31]. This mechanism underlies the strong anticancer activity of withaferin A observed in breast, lung, and brain tumors [27,32].

Withanolides from *W. somnifera* modulate key proteins involved in inflammatory responses and oxidative stress, including PTGS2 (cyclooxygenase-2), JUN, TNF, NF-κB, and NFE2L2 (Nrf2) [29,34]. The expression of these genes is regulated through inhibition of NF-κB activation and activation of the transcription factor Nrf2, leading to increased production of antioxidant enzymes such as superoxide dismutase (SOD), catalase, and heme oxygenase-1 (HO-1) [29,35]. In models of neuroinflammation, ashwagandha reduces levels of pro-inflammatory cytokines (IL-1β, IL-6, TNF-α) in the hippocampus and attenuates microglial activation [36].

Ashwagandha also influences MAPK signaling pathways (ERK1/2, JNK, p38), microRNA regulation in cancer, and cAMP signaling, thereby modulating cell proliferation, apoptosis, and immune responses [32,34]. In models of Huntington’s disease, *W. somnifera* extracts regulate the expression of microRNAs involved in neurogenesis and apoptosis, leading to improvements in cognitive and motor function [36]. Withanolide A induces neurite outgrowth in healthy cortical neurons through activation of the ERK1/2 pathway and increased expression of cytoskeletal proteins [37].

Studies have demonstrated that withanolides act as inhibitors of phosphodiesterase-4D (PDE4D), which may explain their antidepressant and anxiolytic effects via increased intracellular cAMP levels and activation of the PKA/CREB signaling pathway [38]. Inhibition of PDE4D enhances noradrenergic and serotonergic neurotransmission in limbic brain regions, providing a mechanistic basis for the clinical efficacy of ashwagandha in reducing anxiety and depression [38,39].

At the level of the hypothalamic–pituitary–adrenal (HPA) axis, ashwagandha modulates the expression of opioid receptors (MOP and NOP), which is associated with prolonged analgesic effects of morphine and reduced development of opioid tolerance [40]. Standardized root extracts of *W. somnifera* alter basal and morphine-induced expression of opioid receptor genes in neuroblastoma cells, affecting signaling pathways involved in analgesia and addiction [40]. In randomized clinical trials, ashwagandha reduced serum and salivary cortisol levels by 14–28%, correlating with a reduction in symptoms of chronic stress [26,39].

*W. somnifera* influences RNA transcription, stability, and processing, thereby modulating molecular changes associated with aging, including alterations in gene expression and signaling networks [41]. Withanolides regulate genes involved in cell proliferation, apoptosis, immune responses, and energy metabolism through effects on transcription factors such as NF-κB, AP-1, p53, and FOXO [41]. In aging models, ashwagandha increases the expression of genes related to DNA repair, autophagy, and cellular stress resistance, leading to lifespan extension and improved cognitive function [41].

Clinically, ashwagandha demonstrates adaptogenic effects in randomized, double-blind, placebo-controlled trials, reducing stress symptoms (28–44%) and anxiety (41–56%) and improving sleep quality (41–72%) at doses of 240–600 mg/day administered for 8–12 weeks [26,39]. In studies involving athletes, ashwagandha enhances muscle strength, endurance, and recovery through modulation of testosterone, cortisol, and creatine kinase levels [28,39]. Its antidiabetic effects include improved insulin sensitivity and reductions in fasting glucose and HbA1c levels through activation of AMPK and inhibition of gluconeogenesis [42].

### 3.4. Eleutherococcus senticosus (Rupr. & Maxim.) Maxim

**Figure 4:**
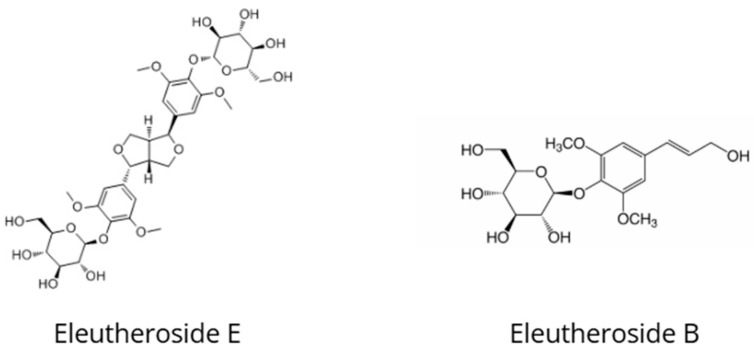
Chemical structures of eleutheroside E and eleutheroside B, characteristic phenolic glycosides present in *Eleutherococcus senticosus*.

*Eleutherococcus senticosus*, commonly known as Siberian ginseng, is a traditional adaptogen that has been used for over two thousand years in Chinese and Russian medicine to reduce fatigue, enhance immune function, and improve physical and mental endurance (Figure 4) [43,44]. The European Medicines Agency (EMA) has approved *E. senticosus* root for the treatment of symptoms of asthenia, such as fatigue and weakness [43]. Eleutheroside E (syringaresinol diglucoside) and eleutheroside B (syringin) are the principal phenolic bioactive constituents and are used as markers for the standardization of *Eleutherococcus* preparations [43,45].

Eleutheroside E, the main phenolic active compound, exhibits a chemical structure similar to catecholamines—mediators of the sympathoadrenal system (SAS) involved in early activation of the stress response [1,43]. This structural analogy to adrenaline and noradrenaline explains the rapid adaptogenic effects of eleutherosides in modulating neuroendocrine responses to acute stressors [1]. Eleutheroside E acts through G-protein-coupled receptor (GPCR) pathways, modulating cyclic AMP (cAMP), phospholipase C (PLC), and phosphatidylinositol signaling cascades [4].

Clinical studies have demonstrated that *Eleutherococcus* improves mental performance, increases adrenal cortical activity, and enhances metabolic intensity under both normal and non-stress conditions [1,43]. Supplementation with *E. senticosus* root extract (300–1200 mg/day) for 4–8 weeks significantly increases physical performance and VO_2_max and reduces fatigue in athletes and individuals with chronic fatigue [43]. These effects involve enhanced muscular glucose utilization, improved mitochondrial biogenesis, and activation of the AMPK signaling pathway [46].

Under stress conditions, *Eleutherococcus* reduces adrenal cortex and sympathetic nervous system activity, increases parasympathetic tone, and moderately enhances central nervous system excitation through modulation of GABAergic and glutamatergic neurotransmission [1,43]. This bidirectional regulatory effect on the hypothalamic–pituitary–adrenal (HPA) axis is characteristic of adaptogens and distinguishes them from conventional stimulants [1]. Eleutheroside B inhibits the activation of MAP kinases (MAPKs), Akt, and NF-κB, leading to reduced levels of pro-inflammatory cytokines IL-1β, IL-6, and TNF-α in models of neuroinflammation [35,43].

The neuroprotective activity of *E. senticosus* includes increased expression of brain-derived neurotrophic factor (BDNF), which promotes neurogenesis and synaptogenesis in the hippocampus [43]. Eleutheroside B exhibits strong antioxidant properties through activation of the Nrf2 pathway and upregulation of antioxidant enzymes, including superoxide dismutase (SOD), catalase, and glutathione peroxidase [43,47]. The antioxidant mechanism of eleutherosides E1 involves both direct free radical scavenging and stimulation of gut-associated lymphoid tissue (GALT), which explains their immunomodulatory effects [47].

*E. senticosus* modulates lipid and glucose metabolism by inhibiting serum superoxide dismutase activity, resulting in increased HDL-C levels and reductions in triglycerides, total cholesterol, and LDL-C in D-galactose-induced aging models [47]. Eleutheroside B inhibits cyclooxygenase-2 (COX-2) through hydrogen bonding with amino acid residues SER530, TYR355, and LEU352, as well as hydrophobic interactions, providing a molecular basis for its anti-inflammatory and analgesic effects [48]. In silico studies have shown that eleutheroside B possesses drug-like properties and does not exhibit significant toxicity (toxicity class 5), hepatotoxicity, or cardiotoxicity [48].

Caffeoylquinic acids, syringin (eleutheroside B), and syringaresinol derivatives are the main constituents responsible for the pharmacological effects of *E. senticosus* [43]. These compounds act synergistically by activating gene sets that are not affected by individual components alone, a hallmark of multicomponent adaptogenic preparations [4]. Studies in honeybees have demonstrated that *E. senticosus* root extract (0.4 mg/mL) is one of the most effective adaptogens in enhancing immune resistance and improving survival in nosemosis [44].

Clinically, *E. senticosus* reduces symptoms of asthenia by 25–40% compared with placebo, improves quality of life, and lowers serum cortisol levels after 4–8 weeks of supplementation [43]. Its immunomodulatory effects include increased activity of natural killer (NK) cells, T lymphocytes, and interferon-γ production, supporting its use in the prevention and treatment of viral respiratory infections [49]. The antiviral mechanisms of *E. senticosus* involve inhibition of viral replication, attenuation of inflammatory progression, and support of recovery through detoxification and repair of oxidative damage [49].

### 3.5. Panax ginseng C.A. Meyer

**Figure 5:**
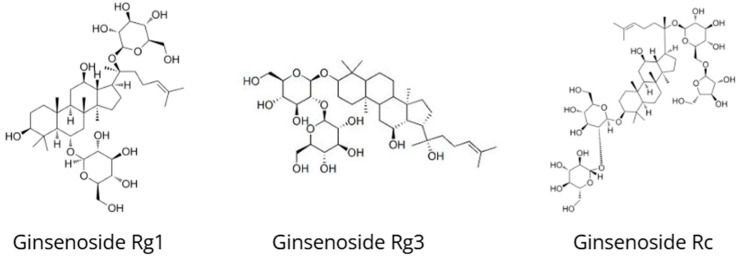
Chemical structures of ginsenoside Rg1, ginsenoside Rg3, and ginsenoside Rc, representative triterpenoid saponins occurring in *Panax ginseng*.

*Panax ginseng*, also known as Korean or Asian ginseng, is one of the most important adaptogens used in Traditional Chinese Medicine for more than 2000 years to strengthen Qi, enhance vitality, and treat stress-related disorders (Figure 5) [50]. *Panax ginseng* contains ginsenosides (triterpenoid saponins) as its principal bioactive constituents responsible for adaptogenic activity. More than 40 different ginsenosides have been identified, including Rb1, Rg1, Rg3, Rc, Rd, and Re [51,52,53]. Ginsenosides are classified into two main groups: protopanaxadiol (PPD; e.g., Rb1, Rc, Rd) and protopanaxatriol (PPT; e.g., Rg1, Re), which exhibit distinct and sometimes opposing pharmacological effects [50].

Ginsenosides Rc and Rg3 and related compounds modulate the hypothalamic–pituitary–adrenal (HPA) axis through inhibition of FK506-binding protein 51 (FKBP51) at the glucocorticoid receptor (GR), resulting in a reduction in depressive-like behaviors in mice exposed to chronic unpredictable mild stress (CUMS) [54]. FKBP51 negatively regulates GR sensitivity; its inhibition by ginsenosides enhances nuclear translocation of the glucocorticoid–GR complex, leading to normalization of HPA axis activity and attenuation of hypercortisolemia [54]. Ginsenoside Rg1 acts as a functional ligand of the glucocorticoid receptor, exerting both genomic and non-genomic effects via GR binding and induction of angiogenesis [55,56].

Total saponins from *P. ginseng* exert antidepressant effects by suppressing the CX3CL1/CX3CR1 signaling axis and inhibiting phosphorylation of the mitogen-activated protein kinases p38 and JNK in the hippocampus of CUMS-exposed rats [57]. CX3CL1 (fractalkine) is a neuron-derived chemokine that, through interaction with CX3CR1 on microglia, activates inflammatory cascades leading to excessive microglial activation and release of pro-inflammatory cytokines such as IL-1β, IL-6, and TNF-α [57]. Total saponins inhibit this axis, thereby reducing neuroinflammation and restoring monoaminergic neurotransmitter balance (serotonin, noradrenaline, dopamine) in the brain [57].

The neuroprotective mechanisms of ginseng include modulation of the GABA–benzodiazepine (GABA-BZD) receptor system, which protects against oxidative stress, mitochondrial dysfunction, and neuroinflammation induced by sleep deprivation in 72 h sleep deprivation models [58]. *Panax quinquefolius* (American ginseng) increases the expression of GABA_A_ receptor subunits (α1, α2, β2) and reduces levels of ROS, malondialdehyde (MDA), and nitric oxide (NO) in the cerebral cortex and hippocampus [58]. This GABAergic mechanism explains the anxiolytic and calming effects of ginseng without the sedative side effects characteristic of benzodiazepines [58].

Ginsenoside Rc enhances mitochondrial biogenesis by increasing the activity of peroxisome proliferator-activated receptor gamma coactivator 1α (PGC-1α), resulting in increased ATP synthesis and reduced reactive oxygen species (ROS) production in skeletal muscle cells [59]. PGC-1α is a master regulator of mitochondrial biogenesis, activating genes encoding mitochondrial proteins, including respiratory chain complexes I–V and ATP synthase [59]. This mechanism accounts for the anti-fatigue and ergogenic effects of ginseng in oxidative stress-induced muscle atrophy [59].

20(S)-Protopanaxadiol (20S-PPD), the major intestinal metabolite of ginsenosides, alleviates DRP1 (dynamin-related protein 1)-dependent mitochondrial dysfunction in models of depression via the SIRT1/PGC-1α signaling pathway [60]. 20S-PPD inhibits excessive mitochondrial fragmentation by reducing DRP1 translocation to mitochondria, thereby improving mitochondrial membrane potential (ΔΨm), decreasing apoptosis, and increasing serotonin (5-HT) levels in neurons [60]. Total ginsenosides from ginseng root regulate the AMPK/SIRT1/PGC-1α pathway, as demonstrated by network pharmacology and transcriptomic analyses, identifying this mechanism as central to their antidepressant effects [61].

Korean red ginseng, produced by steaming and drying fresh *P. ginseng* roots, prevents post-traumatic stress disorder (PTSD)-like depression through activation of the serotonergic system in single prolonged stress (SPS) rat models [62]. It increases mRNA expression of tryptophan hydroxylase-1 (TPH1) in the intestine and tryptophan hydroxylase-2 (TPH2) in the brain, the rate-limiting enzymes of serotonin biosynthesis [62]. Additionally, it elevates serotonin levels in serum and brain tissue and normalizes depressive-like behaviors in forced swimming and open-field tests [62].

Ginsenosides, particularly Rg1, demonstrate strong antidepressant activity in preclinical studies, reducing immobility time in the forced swimming test by 30–50% compared with controls [53]. A meta-analysis of 29 preclinical studies showed that ginsenosides increase the expression of brain-derived neurotrophic factor (BDNF) and its receptor TrkB in the hippocampus and prefrontal cortex, thereby promoting neurogenesis and synaptogenesis [53,62]. Ginsenoside Rd exerts antidepressant effects via activation of the hypoxia-inducible factor 1α (HIF-1α) pathway, increasing the expression of synapsin I (SYN1) and postsynaptic density protein 95 (PSD95), markers of synaptic plasticity [63].

Ginsentides, recently identified cyclic peptides from *P. ginseng*, are key bioactive constituents responsible for the so-called “cure-all” effects of ginseng, coordinating multiple physiological systems to alleviate stress and stress-related disorders [64]. Ginsentides modulate membrane receptors, including opioid, adrenergic, and serotonergic receptors, and exhibit stronger adaptogenic activity than ginsenosides alone [64]. Clinically, hydroponically grown red *P. ginseng* (600 mg/day for 8 weeks) reduced perceived stress by 23%, improved cognitive function, and modulated emotional processing in moderately stressed individuals in a randomized, double-blind, placebo-controlled trial [65].

Ginseng normalizes HPA axis function by regulating the expression of ACTH, corticosterone, and BDNF genes in the hippocampus of CUMS-exposed rats, as demonstrated in molecular mechanistic studies [66]. The standardized ginseng extract G115^®^ enhances the antidepressant effects of fluoxetine by reducing immobility time in the forced swimming test and increasing BDNF expression in the left hippocampus and left prefrontal cortex [64]. Clinically, ginseng is used in the management of cardiovascular diseases, diabetes, rheumatoid arthritis, osteoporosis, erectile dysfunction, and allergic asthma through regulation of the HPA axis and the sympathetic nervous system [67].

### 3.6. Ocimum tenuiflorum L.

**Figure 6:**
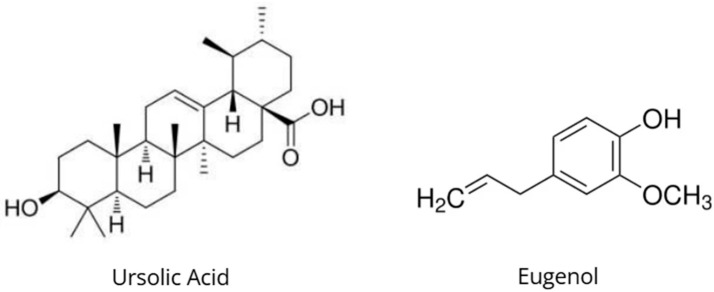
Chemical structures of ursolic acid and eugenol, representative bioactive compounds occurring in *Ocimum sanctum*.

*Ocimum tenuiflorum* L., commonly known as tulsi or holy basil, is one of the most highly valued plants in Ayurveda and traditional Indian medicine due to its broad spectrum of therapeutic activities (Figure 6) [68]. Tulsi is traditionally classified as a *rasayana* (rejuvenating tonic) and an adaptogen, used to enhance resistance to physical, chemical, and biological stressors; improve endurance; and promote longevity [68,69]. *O. tenuiflorum* contains more than 150 bioactive compounds, including phenylpropanoids (eugenol, methyleugenol), triterpenoids (ursolic acid, oleanolic acid), flavonoids (orientin, vicenin), and terpenes (α-pinene, β-caryophyllene), which act synergistically [68].

*O. tenuiflorum* exerts adaptogenic effects through multilevel modulation of the hypothalamic–pituitary–adrenal (HPA) axis, as demonstrated in preclinical *in vivo* and *in vitro* studies [68]. An extract of *O. tenuiflorum* (Holixer^®^, standardized to 2.5% ursolic acid) exhibits antagonistic activity toward corticotropin-releasing factor receptor 1 (CRF1), leading to inhibition of cortisol release and attenuation of HPA axis activation under stress conditions [68]. In an *in vitro* study using HEK293 cells transfected with the human CRF1 receptor, *O. tenuiflorum* extract (1–100 μg/mL) showed statistically significant CRF1 antagonistic activity (IC_50_ = 45.2 μg/mL), inhibiting CRF binding and receptor activation [68].

CRF1 antagonism accounts for increased swimming time in mice (by 56% compared with controls), reduced immobility time (by 38%), and prevention of corticosterone elevation in rats subjected to the forced swimming test following administration of *O. tenuiflorum* extract (50–100 mg/kg body weight) [68]. These behavioral effects indicate antidepressant and anxiolytic activity of tulsi through normalization of the HPA axis and reduction in hypercortisolemia characteristic of chronic stress [68]. In a mouse endurance swimming model, *O. tenuiflorum* extract prolonged time to exhaustion by 92% compared with controls, confirming its anti-fatigue and adaptogenic properties [68].

The anti-stress effects of tulsi are associated with inhibition of corticotropin-releasing hormone (CRH) release at the hypothalamic level and reduced HPA axis activity via negative feedback mechanisms under stress conditions [68]. CRH released from the paraventricular nucleus (PVN) of the hypothalamus stimulates adrenocorticotropic hormone (ACTH) secretion from the pituitary, which in turn increases the synthesis and release of stress glucocorticoids (cortisol in humans and corticosterone in rodents) from the adrenal cortex. Antagonism of CRF1 by *O. tenuiflorum* interrupts this cascade at the pituitary level [69]. In addition, tulsi enhances parasympathetic nervous system activity, thereby reducing sympathoadrenal system (SAS) activation and promoting relaxation and recovery [70].

In vitro studies have confirmed that *O. tenuiflorum* extract directly inhibits cortisol release from H295R adrenocortical cells following stimulation with forskolin (an adenylyl cyclase activator) in a dose-dependent manner (1–100 μg/mL), reducing cortisol levels by 42% at the highest tested concentration [68]. This finding indicates a dual mechanism of action: a central effect (CRF1 antagonism) and a peripheral effect (direct inhibition of adrenal steroidogenesis) [68]. The reduction in cortisol release is mediated by decreased expression of key steroidogenic enzymes, including CYP11A1 (cholesterol side-chain cleavage enzyme) and 3β-hydroxysteroid dehydrogenase (3β-HSD) [68].

Eugenol (4-allyl-2-methoxyphenol), the major constituent of *O. tenuiflorum* essential oil (70–80% of its composition), exhibits strong antioxidant activity through activation of the Nrf2 signaling pathway and upregulation of antioxidant enzymes such as superoxide dismutase (SOD), catalase, and glutathione peroxidase (GSH-Px) [35]. Eugenol suppresses oxidative stress in neurons via direct scavenging of reactive oxygen species (ROS) and modulation of mitochondrial function, thereby protecting against neurodegeneration [36]. Furthermore, eugenol exerts anti-inflammatory effects by inhibiting NF-κB and cyclooxygenase-2 (COX-2) activation, leading to reduced production of pro-inflammatory cytokines IL-1β, IL-6, and TNF-α [35].

Ursolic acid, a pentacyclic triterpenoid from *O. tenuiflorum* leaves, exhibits neuroprotective effects through modulation of apoptotic pathways and increased expression of brain-derived neurotrophic factor (BDNF) and neurotrophin-3 in the hippocampus [71]. Ursolic acid inhibits activation of caspase-3 and caspase-9, prevents loss of mitochondrial membrane potential (ΔΨm), and increases the Bcl-2/Bax ratio, thereby protecting neurons against oxidative stress-induced apoptosis [71]. This mechanism explains the antidepressant effects and cognitive improvements observed following *O. tenuiflorum* supplementation [71].

Clinically, *O. tenuiflorum* (300–600 mg/day of leaf extract for 60 days) reduces symptoms of generalized anxiety by 35–58% compared with placebo in randomized controlled trials [69]. Tulsi decreases serum cortisol levels by 18–24%, improves psychological stress parameters (Perceived Stress Scale, PSS), and enhances sleep quality and overall well-being [69]. In individuals exposed to occupational stress, tulsi supplementation (400 mg/day for 6 weeks) improved memory, attention, and concentration by 20–30% compared with baseline values [69].

*O. tenuiflorum* also exhibits immunomodulatory activity by increasing T- and B-lymphocyte proliferation, natural killer (NK) cell activity, and production of Th1 cytokines (IFN-γ, IL-2), thereby strengthening cell-mediated immunity [69]. In addition, tulsi shows hepatoprotective, cardioprotective, and antidiabetic effects through modulation of the AMPK, PI3K/Akt, and PPAR-γ signaling pathways [69]. Traditional Ayurvedic uses of *O. tenuiflorum* include the treatment of asthma, bronchitis, malaria, diarrhea, dyspepsia, and skin diseases, which are supported by contemporary pharmacological studies [69].

### 3.7. Bacopa monnieri (L.) Westtst.

**Figure 7:**
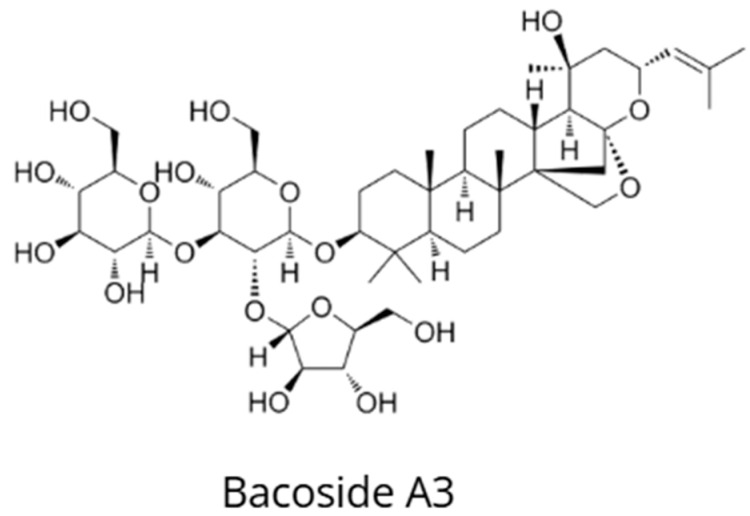
Chemical structure of bacoside A3, a representative triterpenoid saponin and one of the principal bioactive constituents of *Bacopa monnieri*.

*Bacopa monnieri* (L.), also known as brahmi, jalabrahmi, or water hyssop, is one of the most important nootropic plants in Ayurvedic medicine. It has been traditionally used for more than 3000 years as a *medhya rasayana* (cognitive rejuvenating tonic) to enhance memory, learning, and concentration, and to treat anxiety, epilepsy, and neurological disorders (Figure 7) [72,73]. *B. monnieri* contains bacosides A and B as its principal bioactive constituents responsible for neuroprotective and cognitive effects. These compounds are mixtures of dammarane-type triterpenoid saponins containing sugar moieties linked to a steroidal aglycone backbone [74,75]. Bacoside A consists of bacoside A3, bacopasaponin C, bacopasides II and X, and jujubogenin derivatives [74,75].

The mechanism of action of *B. monnieri* involves enhancement of acetylcholine synthesis through activation of choline acetyltransferase (ChAT), the key enzyme catalyzing the biosynthesis of acetylcholine from acetyl-CoA and choline in cholinergic neurons. This leads to improved cholinergic neurotransmission in the brain, particularly in the hippocampus and cerebral cortex [72,73]. In addition, *Bacopa* inhibits acetylcholinesterase (AChE), the enzyme responsible for acetylcholine degradation in the synaptic cleft, thereby prolonging acetylcholine action and strengthening cholinergic signaling [72,76]. In silico and *in vitro* studies have shown that aglycones of bacoside A (jujubogenin and pseudojujubogenin) bind to the active site of AChE (IC_50_ = 4.2–12.8 μM), exhibiting stronger inhibitory activity than bacoside A itself, suggesting *in vivo* conversion to more active forms [74,76].

*B. monnieri* exhibits strong antioxidant properties by increasing the activity of superoxide dismutase (SOD), catalase, and glutathione peroxidase (GPx) in the hippocampus, cerebral cortex, and cerebellum, thereby protecting neurons against oxidative stress-induced damage [72,73]. Its antioxidant mechanism involves activation of the transcription factor Nrf2 (nuclear factor erythroid 2–related factor 2), which induces the expression of genes encoding antioxidant enzymes and phase II detoxification proteins [72]. Administration of *B. monnieri* extract (40–80 mg/kg body weight for 30–60 days) reduces oxidative stress markers, including malondialdehyde (MDA), protein carbonyls (PC), and reactive oxygen species (ROS), while increasing reduced glutathione (GSH) levels by 30–50% in the brains of rats exposed to oxidative stress [72,76].

Bacoside A modulates levels of neurotransmitters, including serotonin (5-HT), dopamine (DA), noradrenaline (NA), acetylcholine (ACh), and GABA in limbic brain structures, contributing to its anxiolytic and antidepressant effects [72,73]. *B. monnieri* increases serotonin levels in the hippocampus, prefrontal cortex, and striatum through inhibition of monoamine oxidase (MAO) and upregulation of serotonergic receptors 5-HT_1_A and 5-HT_2_A, thereby modulating neuronal plasticity underlying memory formation [73]. In silico studies have demonstrated that bacoside aglycones bind with high affinity to 5-HT_1_A, 5-HT_2_A, D_1_, D_2_, and M_1_ receptors (binding energies ranging from −8.2 to −10.5 kcal/mol), explaining the multitarget pharmacological activity of *B. monnieri* [74].

Furthermore, *B. monnieri* promotes neurogenesis by increasing the expression of brain-derived neurotrophic factor (BDNF) and neurotrophin-3 (NT-3) in the hippocampus, thereby supporting neuronal differentiation, growth, and survival [72,75]. Bacoside A activates the ERK1/2 (extracellular signal-regulated kinase) signaling pathway, a member of the mitogen-activated protein kinase (MAPK) family, which regulates cell proliferation, neuronal differentiation, and long-term potentiation (LTP) in the hippocampus [72,73]. ERK1/2 activation leads to phosphorylation of the transcription factor CREB (cAMP response element–binding protein), inducing the expression of genes associated with synaptic plasticity, including BDNF, synapsin I, and Arc [72].

*B. monnieri* reduces β-amyloid (Aβ) accumulation in the brain through multiple mechanisms: enhancement of Aβ-degrading enzyme activity (neprilysin and insulin-degrading enzyme), reduction in Aβ aggregation, and modulation of amyloid precursor protein (APP) processing toward the non-amyloidogenic α-secretase pathway [72,76]. In Alzheimer’s disease models, *B. monnieri* extract (40 mg/kg for 60 days) reduces Aβ_1_–_42_ levels by 35–45% in the hippocampus and cerebral cortex while improving cognitive performance in the Morris water maze and object recognition tests [72,76]. Bacoside A also inhibits tau protein (MAPT) phosphorylation through suppression of glycogen synthase kinase-3β (GSK-3β), thereby preventing the formation of neurofibrillary tangles characteristic of tauopathies [72].

The neuroprotective mechanism of *B. monnieri* also includes enhancement of cerebral blood flow (CBF) via induction of nitric oxide (NO) production in vascular endothelium and relaxation of cerebral smooth muscle, improving oxygen and glucose delivery to neurons and supporting energy metabolism and cognitive function [72]. Bacoside A modulates the expression of synaptic proteins, including synaptophysin, postsynaptic density protein 95 (PSD-95), and synapsin I, increasing dendritic density and dendritic spine formation in the CA1 and CA3 regions of the hippocampus [72,75].

Clinically, *B. monnieri* (300–450 mg/day of extract standardized to 50–55% bacosides for 12 weeks) improves verbal memory, information processing speed, and executive functions by 20–40% compared with placebo, as demonstrated in meta-analyses of randomized controlled trials in healthy adults [76]. In children with ADHD, *B. monnieri* (225 mg/day for 6 months) reduces symptoms of inattention, impulsivity, and anxiety by 30–50% compared with placebo [76]. In elderly individuals with mild cognitive impairment (MCI), *B. monnieri* supplementation (300 mg/day for 12 weeks) improves Mini-Mental State Examination (MMSE) and Alzheimer’s Disease Assessment Scale–Cognitive Subscale (ADAS-Cog) scores by 15–25% [76].

*B. monnieri* exhibits anxiolytic effects comparable to lorazepam (0.5 mg) in models of generalized anxiety, reducing anxiety symptoms by 40–55% on the Hamilton Anxiety Rating Scale (HAM-A) without sedative effects [72,76]. The anxiolytic mechanism involves modulation of GABA_A_ receptors through bacoside binding to allosteric sites, enhancing GABAergic activity and chloride ion conductance [72]. *B. monnieri* also demonstrates antidepressant effects in the forced swimming test, reducing immobility time by 35–50% through increased brain monoamine levels (5-HT, DA, NA) [73].

The antiepileptic activity of *B. monnieri* involves inhibition of L- and T-type calcium channels and modulation of glutamatergic NMDA receptors, thereby reducing neuronal excitability and protecting against seizures induced by maximal electroshock (MES), pentylenetetrazole (PTZ), and picrotoxin [72,76]. *B. monnieri* also protects against heavy metal-induced neurotoxicity (lead, mercury, arsenic, aluminum) through metal ion chelation, increased expression of heat shock proteins (Hsp70, Hsp90), and activation of autophagy pathways [72,75]. Collectively, these multitarget mechanisms confirm the adaptogenic and nootropic properties of *Bacopa monnieri* in the prevention and treatment of neurodegenerative diseases [75,76].

Comparative analysis indicates that all described adaptogens modulate key signaling pathways involved in the regulation of oxidative stress and cellular stress responses, particularly Nrf2/NF-κB and PI3K/Akt [3,4,8,29,72]. Despite their shared pleiotropic mode of action, they differ in their predominant molecular targets. *Rhodiola rosea* and *Schisandra chinensis* demonstrate pronounced mitoprotective effects, including protection of mitochondrial complexes and induction of Hsp70 expression [8,14,16]. *Withania somnifera* primarily affects transcriptional and enzymatic regulation through modulation of PDE4D and transcription factors such as NF-κB and Nrf2 [38,41]. *Panax ginseng* influences glucocorticoid receptor sensitivity by inhibiting FKBP51 and directly modulating GR activity [54,55]. In contrast, *Bacopa monnieri* exerts a predominant effect on cholinergic neurotransmission via activation of choline acetyltransferase (ChAT) and inhibition of acetylcholinesterase (AChE), resulting in improved cognitive function [72,76].

To facilitate comparison of the dominant molecular mechanisms of the analyzed adaptogens, the key bioactive compounds, molecular targets and signaling pathways described in this section are summarized in Table 1.

## 4. Discussion

The collected evidence indicates that plant adaptogens act through pleiotropic and multiaxial modulation of the organism’s stress response, which clearly distinguishes them from classical drugs targeting single molecular entities [2,3]. A key component of their activity is normalization of hypothalamic–pituitary–adrenal (HPA) axis function and integration of neuroendocrine, immune, and metabolic responses, leading to restoration of homeostatic balance under both acute and chronic stress conditions [2,54,66].

At the molecular level, a common denominator of adaptogenic activity is modulation of the PI3K/Akt, AMPK/SIRT1/PGC-1α, and Nrf2/NF-κB signaling pathways [3,8,29,61,72]. This results in improved mitochondrial function, enhanced efficiency of energy metabolism, and attenuation of oxidative stress and neuroinflammation, which has been demonstrated in both preclinical and clinical models [13,33,53,72]. These mechanisms are directly reflected in the neuroprotective, antidepressant, anxiolytic, and anti-fatigue effects observed in experimental and human studies [26,39,53,69,76].

An important contribution to the long-term effects of adaptogens is the induction of heat shock proteins, particularly Hsp70, and the associated phenomenon of cellular hormesis [1,14]. Induction of Hsp70 and activation of Nrf2-dependent antioxidant responses have been described for Schisandra chinensis and Rhodiola rosea, supporting mitochondrial integrity and proteostasis under conditions of oxidative challenge [8,14,16]. These processes increase cellular resistance to damage and promote maintenance of tissue functional stability. At the same time, adaptogens differ in their molecular points of action, as shown by the selective modulation of PDE4D by withanolides [38], FKBP51 and glucocorticoid receptors by ginsenosides [54,55], or cholinergic neurotransmission by bacosides [72,76].

Network pharmacology studies further confirm that the efficacy of adaptogens arises from the synergistic action of multiple bioactive compounds that collectively modulate complex gene and protein networks, rather than from the activity of single isolated constituents [3,4]. Experimental validation of such synergistic transcriptional effects has been demonstrated for multicomponent adaptogenic preparations [4].

Nevertheless, heterogeneity in extract composition and differences in standardization procedures, dosing regimens, and experimental models remain significant limitations of the available evidence [2,3,26]. Studies differ considerably in the type of plant preparations used, ranging from crude extracts to isolated compounds such as salidroside. This variability complicates direct comparison of experimental outcomes and may obscure potential synergistic interactions among phytochemicals present in whole extracts. Additionally, many preclinical studies rely on acute stress models in animals, which may not fully reflect the chronic stress conditions commonly experienced by human populations.

These inconsistencies highlight the need for further systems-level research and well-designed clinical trials incorporating molecular and functional biomarkers to fully validate the therapeutic potential of adaptogens.

Several limitations of the currently available clinical evidence should also be acknowledged. Many randomized clinical trials investigating adaptogens are characterized by relatively small sample sizes, short intervention periods, and heterogeneity in extract standardization and dosing protocols. In addition, only a limited number of studies simultaneously evaluate molecular biomarkers (e.g., FKBP51 expression or cortisol dynamics) together with clinical outcomes, which makes it difficult to directly confirm the mechanistic pathways proposed in experimental models. These factors should be considered when interpreting the reported clinical effects.

## 5. Conclusions

Available evidence confirms that plant adaptogens constitute a rational tool for modulating the organism’s stress response at the molecular, cellular, and systemic levels. Their activity is based on regulation of the hypothalamic–pituitary–adrenal (HPA) axis and key signaling pathways involved in energy homeostasis, neuroprotection, and control of inflammatory processes, which supports their use in the prevention and adjunctive management of disorders associated with chronic stress.

Differences in molecular targets among individual adaptogens indicate the possibility of targeted selection of plant raw materials depending on the dominant pathophysiological mechanisms, such as hypercortisolemia, neuroinflammation, or mitochondrial dysfunction. In clinical practice, this opens the door for more precise and safer use of adaptogens, both as supportive monotherapy and as components of combination therapies.

At the same time, findings from network-based and omics studies suggest that the efficacy of adaptogens is closely linked to preservation of the phytochemical integrity of extracts, and that a reductionist approach based on single activity markers may underestimate their true biological potential. Therefore, future research should focus on functional rather than purely chemical standardization.

In the context of the development of integrative medicine, adaptogens should be regarded as biologically complex regulators of the stress response rather than substitutes for conventional drugs. However, their full implementation into clinical practice requires further well-designed clinical trials and integration of molecular data with clinical parameters and biomarkers of therapeutic response.

## Figures and Tables

**Table 1:** Summary of adaptogens and their role in stress regulation.

Adaptogen	Main Active Compounds	Primary Molecular Mechanisms	Stress-Related Effects Described in the Study
*Rhodiola rosea*	salidroside, tyrosol, rosavins	activation of PI3K/Akt signaling, Nrf2 activation, SIRT1 induction	reduction in fatigue, neuroprotection, improved cognitive performance
*Schisandra chinensis*	schisandrin B, schisandrin C	induction of Hsp70, modulation of PI3K/AKT/mTOR pathway	mitochondrial protection, reduction of oxidative stress
*Withania somnifera*	withanolides (withaferin A, withanolide A)	inhibition of PDE4D, modulation of NF-κB and Nrf2	reduction in cortisol, anti-inflammatory and neuroprotective activity
*Eleutherococcus senticosus*	eleutheroside B, eleutheroside E	GPCR-mediated signaling, modulation of MAPK and NF-κB	improved physical performance, immunomodulation
*Panax ginseng*	ginsenosides (Rb1, Rg1, Rc, Rd)	modulation of glucocorticoid receptor sensitivity, inhibition of FKBP51	antidepressant activity, regulation of HPA axis
*Ocimum tenuiflorum*	eugenol, ursolic acid	antagonism of CRF1 receptor, activation of Nrf2	reduction in cortisol release, antioxidant effects
*Bacopa monnieri*	bacoside A, bacoside B	activation of choline acetyltransferase, inhibition of acetylcholinesterase	improved memory and cognitive function

## Data Availability

Data sharing does not apply in this article as no datasets have been generated.
